# The IgG glycome of SARS-CoV-2 infected individuals reflects disease course and severity

**DOI:** 10.3389/fimmu.2022.993354

**Published:** 2022-10-18

**Authors:** Sterre L. Siekman, Tamas Pongracz, Wenjun Wang, Jan Nouta, Peter G. Kremsner, Pedro Vieira da Silva-Neto, Meral Esen, Andrea Kreidenweiss, Jana Held, Átila Alexandre Trapé, Rolf Fendel, Isabel Kinney Ferreira de Miranda Santos, Manfred Wuhrer, ImmunoCovid Consortium, Alessandro P. de Amorim

**Affiliations:** ^1^ Center for Proteomics and Metabolomics, Leiden University Medical Center, Leiden, Netherlands; ^2^ Institute of Tropical Medicine, University of Tübingen, Tübingen, Germany; ^3^ Centre de Recherches Medicales de Lambaréné, Lambaréné, Gabon; ^4^ German Center for Infection Research, Deutschen Zentrum für Infektionsforschung (DZIF), Partner Site Tübingen, Tübingen, Germany; ^5^ School of Pharmaceutical Sciences of Ribeirão Preto, University of São Paulo, São Paulo, Brazil; ^6^ School of Physical Education and Sport of Ribeirão Preto, University of São Paulo, São Paulo, Brazil; ^7^ Ribeirão Preto Medical School, University of São Paulo, São Paulo, Brazil

**Keywords:** IgG glycosylation, SARS-CoV-2, COVID-19, anti-spike IgG, total IgG

## Abstract

Immunoglobulin G (IgG) antibodies play an important role in the immune response against viruses such as SARS-CoV-2. As the effector functions of IgG are modulated by *N*-glycosylation of the Fc region, the structure and possible function of the IgG *N*-glycome has been under investigation in relation to divergent COVID-19 disease courses. Through LC-MS analysis we studied both total IgG1 and spike protein-specific IgG1 Fc glycosylation of 129 German and 163 Brazilian COVID-19 patients representing diverse patient populations. We found that hospitalized COVID-19 patients displayed decreased levels of total IgG1 bisection and galactosylation and lowered anti-S IgG1 fucosylation and bisection as compared to mild outpatients. Anti-S IgG1 glycosylation was dynamic over the disease course and both anti-S and total IgG1 glycosylation were correlated to inflammatory markers. Further research is needed to dissect the possible role of altered IgG glycosylation profiles in (dys)regulating the immune response in COVID-19.

## Introduction

The rapid spread of the infectious agent severe acute respiratory syndrome coronavirus 2 (SARS-CoV-2) has caused the first global pandemic in the 21^st^ century that gave rise to extensive hospitalizations and a significant economic burden. While the majority of people infected with SARS-CoV-2 remain asymptomatic, a minority of symptomatic infections require hospitalization due to the development of severe coronavirus disease 2019 (COVID-19). Severe COVID-19 is characterized by a dysregulated immune response, of which the nature remains incompletely understood (1). However, certain demographic risk factors for severe COVID-19 have been identified including higher age, male sex and ethnicity, as well as pre-existing comorbidities such as type II diabetes, COPD and hypertension (2–4). Moreover, both immune cell exhaustion (5) and unusual phenotypes thereof (6), a pro-inflammatory cytokine and chemokine milieu in various body fluids (7), together with proteomics and metabolomics signatures (8) have been shown to characterize severe COVID-19 (9).

The observed inflammatory environment is inherent to severe respiratory viral infections (10), but the peculiar, and often unexpected exacerbation of COVID-19 appears to coincide with immunoglobulin G (IgG) seroconversion (11–13). IgGs play an important role both in the neutralization of viral antigens *via* the fragment antigen binding (Fab) portion, and in mediating effector functions *via* its fragment crystallizable (Fc) moiety (14). The effector functions of IgG are modulated by its *N*-glycosylation. IgGs contain two conserved *N*-glycosylation sites in the Fc region at Asn-297 in each of the constant heavy chain 2 domains. At these sites oligosaccharides (glycans) are attached that are made up of a pentasaccharide core of two *N*-acetylglucosamine (GlcNAc) moieties and three mannose residues. This core can be modified by the addition of a fucose residue (fucosylation), a bisecting GlcNAc residue (bisection) and elongated with up to two antennae, each consisting of a GlcNAc and optionally a galactose (galactosylation), of which the latter may be terminated by a sialic acid (sialylation). These Fc region-associated *N*-glycans affect the affinity of the antibody to its cognate receptors on immune cells and consequently modulate the immune response (14, 15). For example, IgG1 with an afucosylated *N*-glycan attached to its Fc domain has, compared to fucosylated IgG, a greatly increased affinity to the Fcγ-receptor IIIa (FcγRIIIa), which regulates antibody-dependent cell-mediated cytotoxicity (ADCC) (16, 17). IgG glycosylation has thus been under investigation for its potential role in COVID-19 and its potential as an early severity marker (17).

Several studies have found that severe COVID-19 is associated with SARS-CoV-2 spike protein-specific (anti-S) IgG afucosylation (18, 19). For example, it has been shown that afucosylated anti-S IgG enriched from sera of severe COVID-19 patients stimulates pro-inflammatory cytokine production by alveolar-like macrophages *in vitro* (11). Furthermore, in an *in vivo* model, afucosylated IgG immune complexes from COVID-19 patients have been shown to induce inflammation and infiltration of the lungs by immune cells (20). A similar afucosylated pathogen-specific antibody response has likewise been observed in Dengue virus infection, malaria and HIV, as well as in alloimmune settings (21–24). A hypothesis has been proposed that the expression of foreign antigens on host or viral membranes triggers such afucosylated IgG responses (18). Interestingly, SARS-CoV-2 mRNA vaccination of SARS-CoV-2 naïve individuals also induced a transient afucosylated IgG response, yet to a lesser extent than in severely ill COVID-19 patients (25). Another study found anti-S IgG1 to be highly fucosylated and enriched in sialylation following mRNA vaccination against SARS-CoV-2, albeit this group has neither investigated early timepoints nor longitudinal changes (20).

Besides these patterns of anti-S IgG glycosylation, characteristic total IgG glycosylation signatures have been observed as well. For example, severe COVID-19 patients have been shown to display decreased bisection of the total IgG *N*-glycome, compared to mild inpatients (26). Additionally, a case-control study has shown that COVID-19 patients had decreased levels of total IgG fucosylation, sialylation and galactosylation compared to controls (27). Moreover, low levels of total IgG galactosylation and sialylation at diagnosis of SARS-CoV-2 infection have been shown to be predictive of poor prognosis (28).

In a previous longitudinal, prospective observational cohort study of hospitalized COVID-19 patients (29), we found skewed glycosylation patterns characterized by increased bisection and decreased galactosylation and sialylation of anti-S IgG1 (normalized to total IgG1) to be associated with increased COVID-19 severity as well as with markers of inflammation, both at hospitalization and at highest disease severity.

Here, we explored IgG1 glycosylation in two geographically distinct cohorts including outpatients and negative controls through affinity purification of IgG from plasma samples and tryptic digestion followed by liquid chromatography mass spectrometry (LC-MS) analysis. We were able to confirm and expand on previous findings regarding both total and anti-S IgG1 glycosylation in COVID-19 patients in two cohorts that were diverse with regards to geographical origin (Brazil and Germany), days since symptom onset and disease severity. Both total and anti-S IgG1 Fc glycosylation were correlated to inflammatory markers. After correction for known confounders of IgG1 glycosylation using logistic regression analysis, we found that decreased total IgG1 bisection and galactosylation as well as decreased anti-S IgG1 fucosylation and bisection were associated with hospitalization.

## Materials and methods

### Study cohorts

Samples from COVID-19 patients with varying disease severity as well as controls included in this study originated from Tübingen (Germany) and São Paulo (Brazil). The Tübingen cohort consisted of 12 inpatients (COV-HCQ, ClinicalTrials ID: NCT04342221), 10 outpatients (COMIHY, ClinicalTrials ID: NCT04340544) and 107 outpatients that were sampled at a late timepoint (TüCoV) (**Table 1**). In the COV-HCQ and COMIHY cohorts SARS-CoV-2 infection was confirmed using reverse transcription polymerase chain reaction (RT-PCR) tests, while in the TüCoV patients infection was confirmed with either an RT-PCR test or an enzyme-linked immunosorbent assay (ELISA) against anti-S antibodies. The São Paulo cohort was made up of 73 inpatients (of which 68 were treated at Hospital Santa Casa de Misericórdia and 5 at Hospital São Paulo), 20 outpatients, 70 convalescent patients (post-hospitalization) from the AEROBICOVID project (30) and 87 SARS-CoV-2 negative control subjects (**Table 1**). In most (>85%) of the participants from the São Paulo cohort SARS-CoV-2 infection was confirmed using RT-PCR tests (Biomol OneStep Kit/COVID-19-Instituto de Molecular Biology of Paraná-IBMP Curitiba/PR, Brazil). In the remaining participants serology-specific IgM and IgG antibodies tests (SARS-CoV-2 antibody test^®^, Guangzhou Wondfo Biotech Co., Ltd., Guangzhou, China) or immunochromatographic tests were used. All clinical trials were performed according to the principles of the Declaration of Helsinki. Ethical clearance was obtained from the Ethical Committee of the University of Tübingen and the units of the University of São Paulo involved in this study. Written informed consent was obtained for trial participation.

**Table 1:** Patient characteristics. The patient characteristics can be viewed separately for each cohort in supplementary materials (**Tables S2**, **S3**).

	Negative controls (n = 87)	Convalescent patients* (n = 70)	Outpatients (n = 30)	Inpatients (n = 85)	Outpatients (late timepoints) (n = 107)
**Age** *median (1st quartile - 3rd quartile)*	35 (28 - 46)	50 (41 - 55)	44 (33 - 52)	62 (55 - 76)	31 (24 - 53)
**BMI** *median (1st quartile - 3rd quartile)*	26 (23 - 28)	30 (27 - 33)	27 (25 - 30)	28 (25 - 32)	24 (22 - 27)
**Days since onset of symptoms** *median (1st quartile - 3rd quartile)*	–	–	15 (10 - 19)	11 (8 - 15)	121 (106 - 141)
**Date of sample collection** *min - max*	04/05/2020 - 07/08/2020	–	29/04/2020 - 25/06/2020	08/04/2020 - 21/01/2021	28/05/2020 - 04/09/2020
**Male** *n (%)*	35 (40%)	26 (37%)	15 (50%)	52 (61%)	41 (38%)
**Female** *n (%)*	52 (60%)	44 (63%)	15 (50%)	33 (39%)	66 (62%)
**São Paulo** *n (%)*	87 (100%)	70 (100%)	20 (67%)	73 (86%)	0 (0%)
**Tübingen** *n (%)*	0 (0%)	0 (0%)	10 (33%)	12 (14%)	107 (100%)

*Convalescent patients are patients that had been hospitalized, but at the time of sample collection were recovering at home.

### Chemicals, reagents and enzymes

Disodium hydrogen phosphate dihydrate, potassium dihydrogen phosphate, sodium chloride and trifluoroacetic acid were obtained from Merck (Darmstadt, Germany). From Sigma-Aldrich (Steinheim, Germany) ammonium bicarbonate, formic acid, potassium chloride and tolylsulfonyl phenylalanyl chloromethyl ketone-treated trypsin from bovine pancreas were purchased. The Visucon-F pooled healthy human plasma standard was obtained from Affinity Biologicals (Ancaster, Canada). HPLC-supra-gradient acetonitrile originated from Biosolve (Valkenswaard, The Netherlands). From GE Healthcare (Uppsala, Sweden) protein G Sepharose 4 Fast Flow beads were purchased. Recombinant trimerized S protein was prepared as described previously (31). An ELGA Purelab Ultra system (Elga LabWater, High Wycombe, United Kingdom) was used to produce type I Ultrapure Water that was used in solutions throughout.

### Sample preparation

Anti-S IgG was captured through affinity purification with recombinant trimerized S protein-coated Maxisorp NUNC-Immuno plates (Thermo Fisher Scientific, Roskilde, Denmark) (18), while total IgG was enriched using protein G Sepharose Fast Flow 4 beads (32). A 100 mM formic acid solution was used for antibody elution, followed by sample drying through vacuum centrifugation and reconstitution in 25 mM ammonium bicarbonate. The purified antibodies were subjected to tryptic digestion to obtain glycopeptides, as described previously (29, 32). For the samples from Brazil, a minimum of 2 Visucon-F standards, 4 pooled anti-S IgG samples and 2 blanks were included per plate. For the German samples at least 1 Visucon-F standard and 1 blank was included on each plate.

### IgG Fc glycosylation analysis

The obtained glycopeptides were detected with an Impact HD quadrupole time-of-flight mass spectrometer (Bruker Daltonics, Billerica, MA) following separation using an Ultimate 3000 high-performance liquid chromatography (HPLC) system (Thermo Fisher Scientific, Waltham, MA), as described (29, 32). IgG1 glycoforms were assigned on the basis of accurate mass and specific migration positions in liquid chromatography. Other glycoforms were excluded from analysis to circumvent interference of IgG3- with IgG2- and IgG4-glycopeptides due to the potential overlap in the amino acid sequences of allotypic variants (14).

### Cytokine quantification

All cytokines (IL-6, IL-8, IL-1b, IL-10, and TNF) were quantified in heparinized plasma using the BD Cytometric Bead Array Human Inflammatory Kit (BD Biosciences, San Jose, CA) according to the manufacturer’s instructions. Briefly, after sample processing, the cytokine beads were counted using a flow cytometer (FACSCanto II; BD Biosciences, San Jose, CA), and analyses were performed using FCAP Array (3.0) software (BD Biosciences). The concentrations of cytokines were expressed as pg/ml. Cytokines were measured in samples collected at the same timepoint as the samples used in this study for determining IgG1 Fc *N*-glycosylation profiles and infection status for SARS-CoV-2.

### Data processing

Liquid chromatography-mass spectrometry (LC-MS) data were converted into mzXML files. Alignment and targeted extraction of the raw data was performed using the in-house developed software LacyTools (33). LacyTools was first used to align the runs based on the average retention time of a minimum of three abundant IgG1 glycoforms, and second, to perform targeted data extraction. The extraction list consisted of analytes in 2^+^ and 3^+^ charge states (**Table S1**) (29). Repeatability was assessed by measuring a pre-COVID-19 plasma pool (Visucon-F) and, in the case of the Brazilian samples, pooled anti-S IgG samples from patients present in replicates on each plate. Spectra were excluded from further analysis if their sum intensity was below the average sum intensity plus three times the standard deviation of the anti-S IgG1 signal of negative controls. Signals were integrated by covering a minimum of 95% of the area of the isotopic envelope of glycopeptide peaks. Isotopic peaks of a glycopeptide that may have overlapped with contaminants were excluded from integration (**Table S1**).

### Statistical analysis

**Figure 1:**
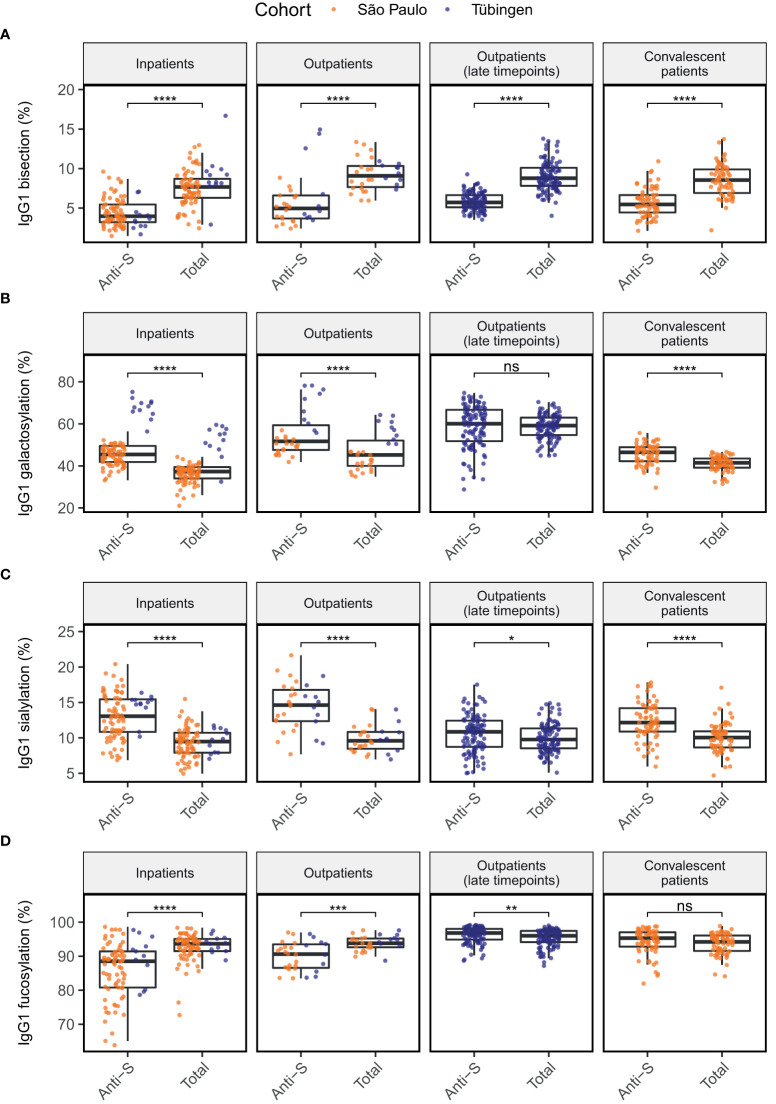
Comparison of anti-S to total IgG1 glycosylation in four patients groups for glycosylation traits bisection **(A)**, galactosylation **(B)**, sialylation **(C)** and fucosylation **(D)**. Significance levels shown are based on the *p*-values from Wilcoxon signed-rank tests. *, **, ***, ****: *p*-value < 0.05, 0.01, 0.001, 0.0001, respectively and ns, not significant (*p-*value ≥ 0.05).

Total area normalization of IgG1 Fc glycopeptides was applied to calculate the relative abundance of each glycoform. The relative abundances of related glycopeptide species were summarized for calculating the glycosylation traits fucosylation, bisection, galactosylation and sialylation, as described previously (29). To compare the anti-S and total IgG1 Fc glycosylation profiles Wilcoxon signed-rank tests were performed (**Figure 1**). Spearman’s correlations were computed to assess the effects of the days since onset of symptoms and of body mass index (BMI) on IgG1 glycosylation and to explore correlations with inflammatory markers (**Figures 3**, **S3**, **6**, **S6**). The comparisons between different biological groups for both total and anti-S IgG1 glycosylation were performed using logistic regression (**Figures 4**, **5**, **S4**, **S5**). For each glycosylation trait a model was built to predict for example whether a patient was hospitalized or not. Age, sex, BMI, the cohort and the interaction between age and sex were included as covariates in all the logistic regression models to adjust for potential confounding effects, whereas the days since symptom onset was included when applicable (**Figures 5**, **S4**, **S5**). The *p*-values of the coefficients corresponding to the glycosylation traits were used to determine whether that particular glycosylation trait was a significant predictor of for example hospitalization and were shown as significance levels in **Figures 4**, **5**, **S4** and **S5**. Odd ratios with 95% confidence intervals can be found in **Tables S6**, **S7**. To explore the confounding effect of the cohort on IgG1 glycosylation Wilcoxon rank sum tests were performed (**Figure S1**).

## Results

In this study, anti-S and total IgG1 Fc glycosylation profiles were characterized of 163 COVID-19 infected patients from a Brazilian and 129 COVID-19 patients from a German cohort. Both cohorts comprised hospitalized (inpatients), convalescent as well as non-hospitalized patients (outpatients). In addition, 87 negative control subjects were included in the Brazilian cohort. The characteristics of the study groups are summarized in **Table 1**.

### Anti-S IgG1 glycosylation differs from total IgG1 glycosylation

To explore how the obtained results align with previous studies, we first compared anti-S IgG1 glycosylation of the patient groups to their total IgG1 glycosylation in a paired manner (**Figure 1**). In all patient groups, glycosylation of anti-S IgG1 was skewed towards low bisection (**Figure 1A**), high galactosylation (**Figure 1B**) and high sialylation (**Figure 1C**) when compared to total IgG1. Anti-S IgG1 was skewed towards low fucosylation in outpatients and in inpatients. In contrast, slightly increased fucosylation characterized the late timepoints of outpatients (**Figure 1D**). In addition, anti-S IgG1 galactosylation at the late timepoints of outpatients (days since onset of symptoms >= 41, median = 121) was similar to total IgG1 galactosylation (**Figure 1B**). No significant difference was found between the anti-S and total IgG1 fucosylation profiles of convalescent patients.

Interestingly, the level of IgG1 galactosylation seemed to be higher overall in patients from the German cohort compared to those from the Brazilian cohort (**Figure 1B**). Upon further examination, we found that there was a significant difference in both anti-S and total IgG1 galactosylation, as well as in total IgG1 bisection and anti-S IgG1 fucosylation between the Brazilian and German patients (**Figure S1**). Because of these observations, we decided to adjust our later analyses not only for the confounding effects of age, sex and BMI, but also for the cohort.

### Anti-S IgG1 glycosylation is dynamic

**Figure 2:**
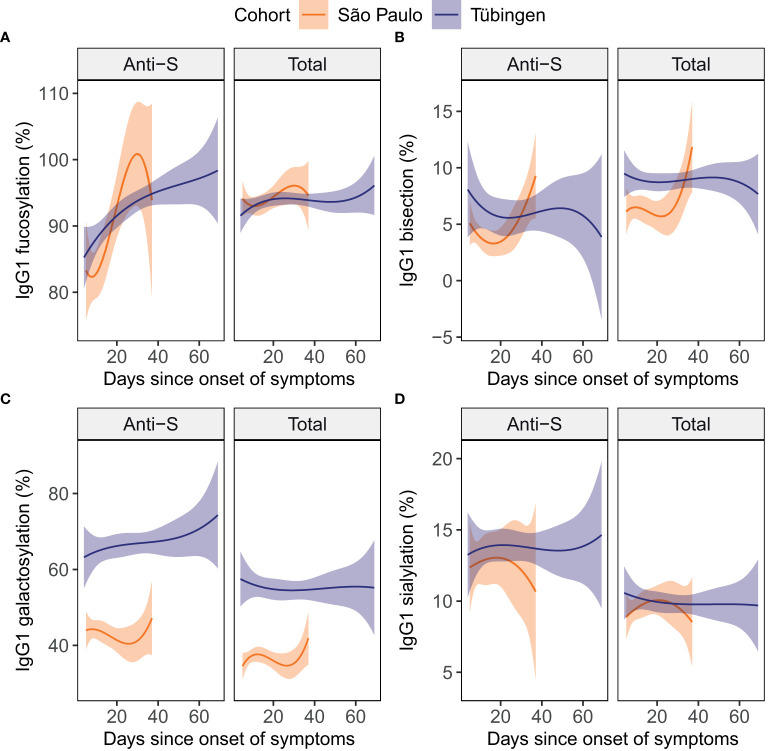
Total and anti-S IgG1 glycosylation in patients over time. For the glycosylation traits fucosylation **(A)**, bisection **(B)**, galactosylation **(C)** and sialylation **(D)** both anti-S (left) and total (right) IgG1 glycosylation are plotted against the days since onset of symptoms. To illustrate the dynamics in each cohort, cubic polynomial curves fitted to the data are shown as lines with 95% confidence intervals shown in orange and purple for the São Paulo (15 inpatients) and the Tübingen (10 inpatients and 10 outpatients) cohort, respectively. Datapoints per individual patient can be viewed in supplementary **Figure S2**.

Various studies have described IgG glycosylation to be dynamic over the course of COVID-19 (11, 18, 29), suggesting that the days since symptom onset could be an important factor in the association of IgG glycosylation with disease severity. Therefore, we studied longitudinal samples of 35 patients to assess the dynamics of IgG1 glycosylation over the COVID-19 disease course. Anti-S IgG1 fucosylation appeared to increase with time (**Figure 2A**). In contrast, total IgG1 glycosylation did not appear to be dynamic over the disease course in these cohorts (**Figures 2A–D**).

**Figure 3:**
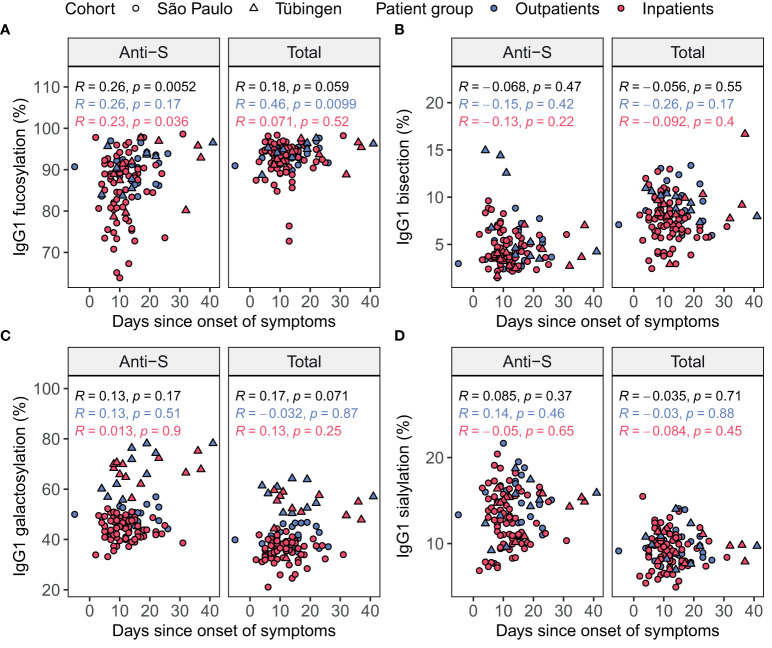
Spearman’s correlation of days since symptom onset and IgG1 glycosylation in patients at home (blue) and inpatients (red) in each cohort. Glycosylation traits fucosylation **(A)**, bisection **(B)**, galactosylation **(C)** and sialylation **(D)** were plotted both for anti-S (left) and total (right) IgG1 Fc glycosylation against the days since onset of symptoms. Brazilian patients are shown as circles, while German patients are shown as triangles. Spearman’s correlation coefficients (*R*) are shown with corresponding *p*-values both separately for the patients at home (blue) and inpatients (red) and for all patients combined (black).

To further explore the potential confounding effect of the sampling day we assessed the Spearman’s correlations between the days since onset of symptoms and the levels of glycosylation traits in baseline samples (**Figure 3**). In the inpatients and outpatients combined, anti-S IgG1 fucosylation was positively correlated to the days since onset of symptoms (**Figure 3A**). In the outpatients a positive correlation was found between the days since onset of symptoms and total IgG1 fucosylation (**Figure 3A**). Therefore, we concluded that the days since onset of symptoms is an additional confounder of IgG1 fucosylation and therefore decided to include it as an additional covariate for later analyses.

### IgG1 glycosylation profiles differ between patient groups and negative controls

**Figure 4:**
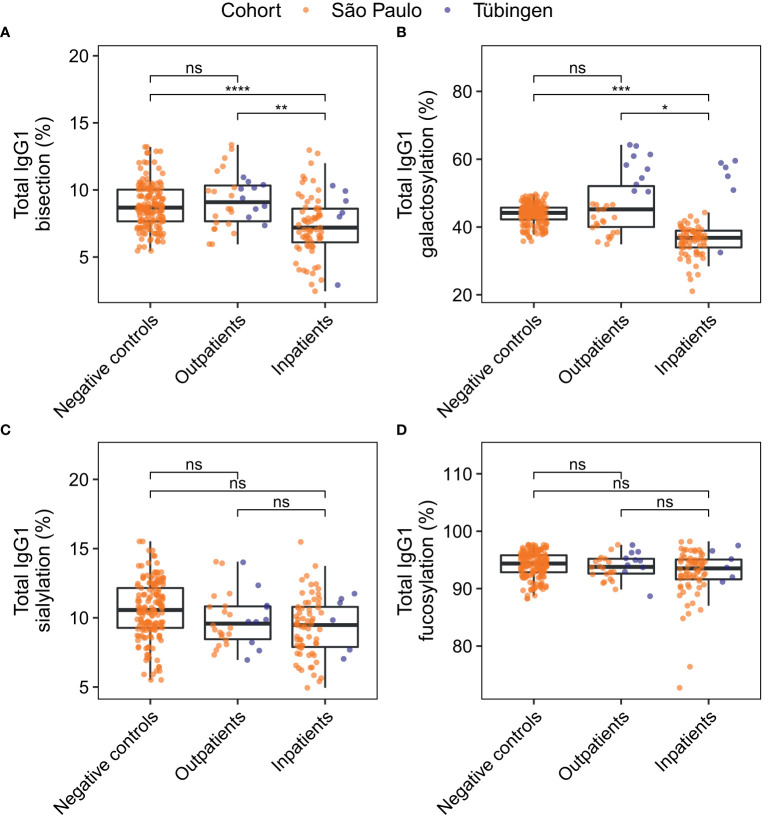
Comparison of total IgG1 glycosylation between patient groups. For the derived glycosylation traits bisection **(A)**, galactosylation **(B)** and sialylation **(C)** and fucosylation **(D)** total IgG1 glycosylation was compared between negative controls (n = 81) and patients at home (n = 30), and between negative controls and inpatients (n = 73). Study participants of whom the BMI was unknown were not included in this analysis. The significance levels shown are based on the *p*-values of the coefficients for the glycosylation traits in the logistic regression models with adjustment for the effects of age, sex, BMI, the cohort and the interaction between age and sex. *, **, ***, ****: *p*-value < 0.05, 0.01, 0.001, 0.0001 and ns: not significant (*p*-value ≥ 0.05). Differences in bisection and galactosylation between outpatients and inpatients remained significant after correction for days since disease onset (**Figure S4**).

Total IgG1 Fc glycosylation profiles of both inpatients and outpatients were compared to those of negative controls through logistic regression analysis. For each glycosylation trait three logistic regression models were built. The first two models predicted the probability of a patient being an outpatient or a hospitalized patient, respectively, rather than a negative control. The third model served to predict the probability of a patient being a hospitalized patient rather than an outpatient. In each model the patients’ age, sex, BMI and the cohort were included as covariates to account for possible confounding effects. Increased BMI is known to be associated with decreased IgG galactosylation (34), which was reflected in these cohorts (**Figure S3**). The differences in the levels of the various glycosylation traits between the patient groups and negative controls are visualized in **Figure 4**. The significance levels shown are based on the *p*-values of the glycosylation traits’ coefficients in the regression models (**Table S6**). These *p*-values give an indication of whether the glycosylation trait has predictive value of the patient group, while taking confounding factors of glycosylation into account. Inpatients were characterized by decreased total IgG1 bisection (**Figure 4A**) and galactosylation (**Figure 4B**) as compared to outpatients and negative controls. In contrast, outpatients were not significantly different from negative controls with regards to their total IgG1 glycosylation (**Figures 4A–D**).

**Figure 5:**
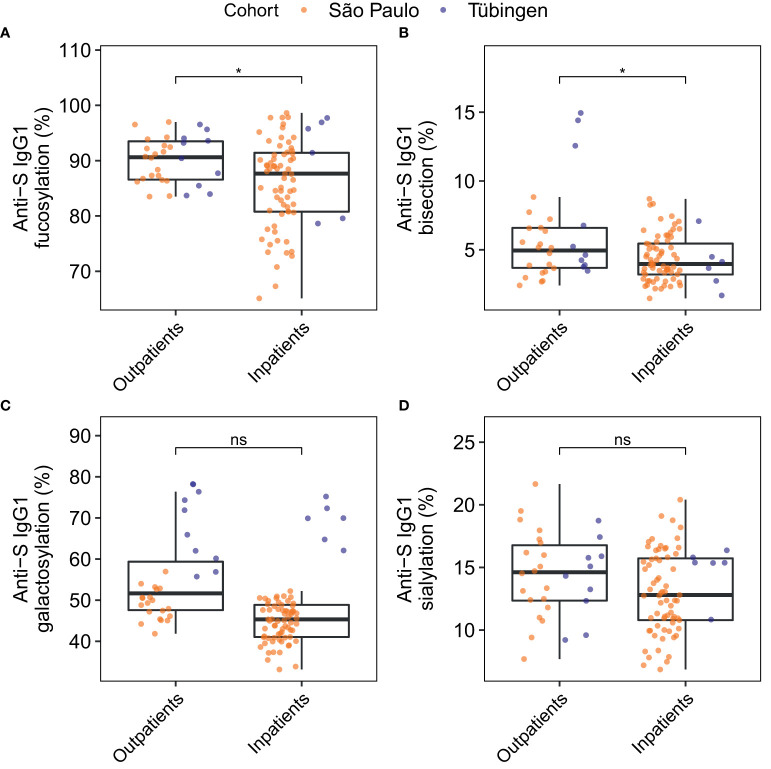
Comparison of anti-S IgG1 glycosylation of inpatients and outpatients for the glycosylation traits fucosylation **(A)**, bisection **(B)**, galactosylation **(C)** and sialylation **(D)**. The significance levels shown are based on the *p*-values of the coefficients for the glycosylation traits in the logistic regression models with adjustment for the effects of age, sex, BMI, the days since onset of symptoms, the cohort and the interaction between age and sex. Study participants of whom the BMI was unknown were not included in this analysis (number of inpatients = 73, number of outpatients = 30). *p-value < 0.05 and ns, not-significant (*p*-value ≥ 0.05).

Next, anti-S IgG1 glycosylation of inpatients was compared to that of outpatients (**Figure 5** and **Table S7** **)**. The levels of anti-S IgG1 fucosylation (**Figure 5A**) and bisection (**Figure 5B**) were slightly decreased in inpatients compared to outpatients. Additionally, anti-S IgG1 glycosylation was compared between patients that had been admitted to an intensive care unit (ICU) and patients that had not been admitted to an ICU (non-ICU) (**Figure S5**). However, no difference was found between the anti-S IgG1 glycosylation of ICU and non-ICU patients in this study.

### Associations between IgG1 glycosylation and cytokine levels

**Figure 6:**
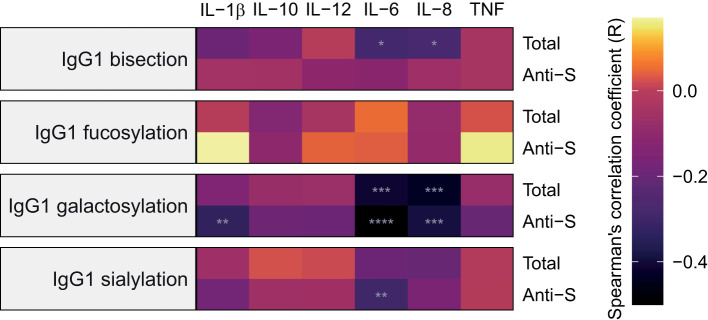
Heatmap of Spearman’s correlations between inflammatory markers and IgG1 glycosylation traits at baseline. Anti-S IgG1 glycosylation is shown on the bottom part of each panel and total IgG1 glycosylation on the top for 57 inpatients and 20 outpatients from the São Paulo cohort. Cytokines were measured in samples collected at the same timepoint as the samples used for determining IgG1 Fc *N*-glycosylation profiles and infection status for SARS-CoV-2. The significance levels indicated with grey asterisks are based on the *p*-value of the Spearman’s correlation coefficient (*R*). *, **, ****: *p*-value < 0.05, 0.01, 0.0001, respectively.

The concentrations of various cytokines including several interleukins (ILs) and tumor necrosis factor alpha (TNFα) at baseline were available for 57 inpatients and 20 outpatients from the Brazilian cohort. We studied the associations between IgG1 Fc glycosylation and cytokine levels using Spearman’s correlations (**Figures 6**, **S6**; **Table S8**). The concentration of the proinflammatory cytokine IL-6 was negatively correlated to anti-S and total IgG1 galactosylation and sialylation as well as to total IgG1 bisection. The cytokine IL-8 showed a similar pattern of correlations to that of IL-6, except for a lack of significant correlation with anti-S IgG1 sialylation. In addition, the concentration of the proinflammatory cytokine IL-1β correlated negatively with anti-S IgG1 galactosylation.

## Discussion

In this study we determined both the total and anti-S IgG1 Fc glycosylation profiles of 163 Brazilian and 129 German COVID-19 patients. This cohort represents the full spectrum of COVID-19 disease severities, comprising inpatients with varying disease severity, large groups of outpatients at different times during and after their illness as well as convalescent patients. The reliability of our results was bolstered by adjusting for known confounders of glycosylation including age, sex (and their interaction) and BMI, as well as for the cohort (country of residence) and time since symptom onset, of which the latter was found to be a major confounder of anti-S IgG1 glycosylation in line with a previous study (29). Of note, adjustment for these confounding factors has been lacking in most preceding studies (18–20, 26, 27), despite their strong association with glycosylation. We observed that anti-S IgG1 glycosylation profiles diverged from those of total IgG1 in all patient groups, largely in line with previous reports (18, 29). We found that both the anti-S and total IgG1 glycome of COVID-19 infected individuals reflected disease course and severity.

The observed differences in anti-S IgG1 glycosylation compared to total IgG1 glycosylation were similar to what has previously been reported for inpatients (18, 29) and for a small cohort of outpatients (18). Moreover, we found comparable differences in convalescent patients, except regarding fucosylation, which remained unchanged. Similar to previous reports, we found anti-S IgG1 skewing towards low fucosylation in inpatients to be transient (18, 29). This temporary nature is underlined by the absence of skewing of anti-S IgG1 towards afucosylation in convalescent patients, suggesting that the low levels of anti-S IgG1 fucosylation act as an early inflammatory signal in the transition of mild-to-severe COVID-19 (18, 20). Indeed, we found that anti-S IgG1 afucosylation was significantly increased in inpatients relative to outpatients. Anti-S IgG1 afucosylation has been associated with COVID-19 severity in a number of studies (11, 18–20, 29) and reports using both *in vitro* (11) and *in vivo* (20) experiments have shown that afucosylated anti-S IgG can stimulate pro-inflammatory cytokine production. However, in line with our previous report on anti-S IgG1 Fc glycosylation (29), we observed no negative correlations between anti-S IgG1 fucosylation and inflammatory markers in patients’ plasma, which may merely be due to the fact that fucosylation of the studied circulatory anti-S IgG1 does not represent the fucosylation of IgG in lung tissues in the form of immune complexes with the S protein, that potentially evoke inflammation at an earlier stage of the disease. In contrast to Hou *et al.* (27), we observed no increase in total IgG1 fucosylation in COVID-19 patients compared to controls, nor did we observe an association between total IgG1 fucosylation and disease severity. Alterations of total IgG glycosylation profiles are largely influenced by anti-S levels, a potential additional source of biological variation that may contribute to this contrasting observation in our study.

When compared to outpatients, inpatients were characterized by decreased anti-S and total IgG1 bisection, in agreement with our previous study (29) and with Petrovíc et al., who reported that total IgG bisection was associated with COVID-19 severity (26). Additionally, in a longitudinal observational study total IgG bisection decreased over the COVID-19 disease course in severe, but not in mild and asymptomatic patients (35). A recent study has indicated that bisection may increase the affinity of monomeric IgG to FcγRIIIa (36), albeit little is known about the functional implications of bisection of IgG antibodies *in vivo*.

Contrasting findings surround the functional effect of IgG galactosylation. Decreased IgG galactosylation has been associated with many autoimmune and infectious diseases and has been linked to inflammation *via* several mechanisms (37). In addition, agalactosylated IgG has been suggested to play a role in the activation of complement *via* the alternative pathway and the mannose-binding lectin pathway (37). On the other hand, galactosylation of IgG1 immune complexes has been described to stimulate dectin-1-mediated signaling that leads to phosphorylation of FcγRIIb which inhibits the pro-inflammatory activation of complement (38). Contrary to this, it has recently been shown that galactosylation of IgG promotes hexamerization and thereby enhances complement activation (39). In the context of COVID-19, and in line with previous findings (27, 28), we found that hospitalized COVID-19 patients had decreased levels of total IgG1 galactosylation compared to control subjects and outpatients. Hou *et al.* reported decreased total IgG galactosylation in COVID-19 cases compared to controls (27). Moreover, Vicente *et al.* reported that decreased total IgG galactosylation at diagnosis indicates poor prognosis, and is accompanied by higher NK cell activation (28). These observations and the link between IgG galactosylation and inflammation suggest that decreased galactosylation of IgG may play a role in the inflammation observed in severe COVID-19. In contrast to previous studies (18, 29), we found no association between anti-S IgG1 galactosylation and COVID-19 severity. This discrepancy might partly be explained by our more thorough adjustment for confounding factors. Moreover, in contrast to Vicente *et al.*, we did not find a sialylation signature associated with COVID-19 severity or hospitalization of COVID-19 patients.

Cytokines and chemokines play an important role in inflammatory settings such as COVID-19. For example, IL-6 has been shown as an important marker of COVID-19 severity (7, 40). In addition, IL-8 is a chemoattractant for neutrophils and can induce the generation of neutrophil extracellular traps (NETs), which have been suggested to contribute to organ damage in severely ill COVID-19 patients (41). We observed negative correlations between the pro-inflammatory markers IL-6, IL-8 and IL-1β and IgG1 glycosylation traits galactosylation, bisection and sialylation, in line with our previous study (29).

Glycosylation has been described to be influenced by both genetic and environmental factors (37). Accordingly, glycosylation of IgG varies between different populations, with the largest variations being in the level of galactosylation (42). For example, individuals living in developing countries were shown to have decreased IgG1 galactosylation, which has been associated with immune activation (43), indicating that environmental factors and immune activation may be another plausible cause for the differences in glycosylation, besides genetics (37, 42). Likewise, we observed differences between the Brazilian and German patients with regard to galactosylation, justifying the addition of the cohort as a covariate in our logistic regression model.

In conclusion, this study explored both total and anti-S IgG1 glycosylation profiles of inpatients and outpatients at various times during and after their COVID-19 disease course. Inpatients when compared to outpatients and SARS-CoV-2-negative control subjects were characterized by low total IgG1 galactosylation and bisection as well as low anti-S IgG1 fucosylation and bisection. Anti-S IgG1 glycosylation was dynamic over the disease course, in contrast to total IgG1 glycosylation, but both were correlated with markers of inflammation. This study included large cohorts from two continents, supporting the general validity of our results. Furthermore, we were able to replicate some of the previously reported IgG glycosylation patterns in COVID-19, which we believe to be a valuable step towards possible clinical translation, with the added value of thoroughly accounting for known confounders of both anti-S and total IgG1 glycosylation, while furthering our understanding of the potential role of IgG1 glycosylation in COVID-19 progression.

## ImmunoCovid Consortium (in alphabetical order)

Alessandro P. de Amorim^2^, Jamille G M Argolo^1^, Rita de C.C. Barbieri^3^, Marcelo Dias-Baruffi^5^, Victor A F Bastos^5^, Vânia L D Bonato^4^, Cristina Ribeiro de Barros Cardoso^5^, Ingryd Carmona-Garcia^1^, Jonatan C S de Carvalho^3^, Leticia F Constant^2^, Augusto M Degiovani^2^, Cassia F.S.L. Dias^3^, Lúcia H Faccioli^5^, Marley R Feitosa^4^, Omar Feres^4^, Ana Paula Morais Fernandes^1^, Talita M Fernandes^1^, Thais F C Fraga-Silva^4^, Carlos Fuzo^5^, Isabelle C. Guarneri^1^, Cristiane M. Milanezi^4^, Caroline T. Garbato^1^, Gilberto Gambero Gaspar^4^, Ângelo A.F. Júnior^1^, Sandra R. Maruyama^6^, Debora C. Nepomuceno^2^, Nicola T Neto^1^, Camilla Narjara Simão Oliveira^5^, Fátima M Ostini^2^, Rogerio S Parra^4^, Malena M Pérez^5^, Vinícius E Pimentel^5^, Giovanna da S. Porcel^1^, José J R da Rocha^3^, Lilian C Rodrigues^5^, Elisa M S Russo^1^, Dayane P. da Silva^2^, Rafael C. da Silva^2^, Carlos Arterio Sorgi^7^, Camila O S Souza^5^, Diana M Toro^5^, Angelina L Viana^1^, Fernando Crivelenti Vilar ^4^, Ana C. Xavier^1^, Kamila Zaparoli^1^

*^1^Escola de Enfermagem de Ribeirão Preto, Universidade de São Paulo, Ribeirão Preto, SP, Brazil*

*^2^Hospital Santa Casa de Misericórdia de Ribeirão Preto, Ribeirão Preto, SP, Brazil.*

*^3^Hospital São Paulo, Ribeirão Preto, SP, Brazil.*

*^4^Faculdade de Medicina de Ribeirão Preto, Universidade de São Paulo, Ribeirão Preto, SP, Brazil.*

*^5^Faculdade de Ciências Farmacêuticas de Ribeirão Preto, Universidade de São Paulo, Ribeirão Preto, SP, Brazil.*

*^6^Centro de Ciências Biológicas e da Saúde, Universidade Federal de São Carlos*

*^7^Faculdade de Filosofia, Ciências e Letras de Ribeirão Preto, Universidade de São Paulo, Ribeirão Preto, SP, Brazil.*

## Data availability statement

The original contributions presented in the study are included in the article/**Supplementary Material**. Further inquiries can be directed to the corresponding author.

## Ethics statement

This study was reviewed and approved by the Ethical Committee of the University of Tübingen and the units of the University of São Paulo involved in this study. The patients/participants provided their written informed consent to participate in this study.

## Author contributions

SS: Data (pre-)processing, data curation, formal analysis, writing – original draft preparation, visualization. TP: Data (pre-)processing, data curation, formal analysis, writing – original draft preparation, supervision. WW and JN: Sample preparation and data generation, data quality control. PK, RF, IS: Study design, development of data analysis plan. AT: Study design, acquisition and development of data. PS-N: Acquisition of data and development of data analysis plan. ME, AK, JH: Study design. MW: Funding acquisition, coordination, supervision, data interpretation, writing - review and editing. All authors contributed to the article and approved the submitted version.

## Funding

Supported by grant 2020/05207-6 from the Fundação de Amparo à Pesquisa do Estado de São Paulo (FAPESP) to L.H.F, of the University of São Paulo’s ImmunoCovid Consortium. TP has received funding from the European Union’s Horizon 2020 research and innovation programme, under H2020-MSCA-ITN grant agreement number 721815. The AEROBICOVID project received funding from the ‘USP Vida’ Project (code – 3518/2020) and Integrated Research Projects in Strategic Areas (PIPAE) from the Dean of Research-USP (2021.1.10424.1.9)

## Conflict of interest

The authors declare that the research was conducted in the absence of any commercial or financial relationships that could be construed as a potential conflict of interest.

## Publisher’s note

All claims expressed in this article are solely those of the authors and do not necessarily represent those of their affiliated organizations, or those of the publisher, the editors and the reviewers. Any product that may be evaluated in this article, or claim that may be made by its manufacturer, is not guaranteed or endorsed by the publisher.

## References

[1] Merad M Martin JC. Pathological inflammation in patients with covid-19: A key role for monocytes and macrophages. Nat Rev Immunol (2020) 20(6):355–62. doi: 10.1038/s41577-020-0331-4 PMC7201395 32376901

[2] Williamson EJ Walker AJ Bhaskaran K Bacon S Bates C Morton CE. Factors associated with covid-19-Related death using opensafely. Nature (2020) 584(7821):430–6. doi: 10.1038/s41586-020-2521-4 PMC7611074 32640463

[3] Golestaneh L Neugarten J Fisher M Billett HH Gil MR Johns T. The association of race and covid-19 mortality. EClinicalMedicine (2020) 25:100455. doi: 10.1016/j.eclinm.2020.100455 32838233 PMC7361093

[4] Zhang J-j Dong X Liu G-h Gao Y-d. Risk and protective factors for covid-19 morbidity, severity, and mortality. Clin Rev Allergy Immunol (2022) 62(1):1–18. doi: 10.1007/s12016-022-08921-5 PMC8767775 35044620

[5] Zheng M Gao Y Wang G Song G Liu S Sun D. Functional exhaustion of antiviral lymphocytes in covid-19 patients. Cell Mol Immunol (2020) 17(5):533–5. doi: 10.1038/s41423-020-0402-2 PMC7091858 32203188

[6] Su Y Chen D Yuan D Lausted C Choi J Dai CL. Multi-omics resolves a sharp disease-state shift between mild and moderate covid-19. Cell (2020) 183(6):1479–95.e20. doi: 10.1016/j.cell.2020.10.037 33171100 PMC7598382

[7] Del Valle DM Kim-Schulze S Huang H-H Beckmann ND Nirenberg S Wang B. An inflammatory cytokine signature predicts covid-19 severity and survival. Nat Med (2020) 26(10):1636–43. doi: 10.1038/s41591-020-1051-9 PMC7869028 32839624

[8] Shen B Yi X Sun Y Bi X Du J Zhang C. Proteomic and metabolomic characterization of covid-19 patient sera. Cell (2020) 182(1):59–72. doi: 10.1016/j.cell.2020.05.032 32492406 PMC7254001

[9] Huang W Li M Luo G Wu X Su B Zhao L. The inflammatory factors associated with disease severity to predict covid-19 progression. J Immunol (2021) 206(7):1597–608. doi: 10.4049/jimmunol.2001327 33579725

[10] Camp JV Jonsson CB. A role for neutrophils in viral respiratory disease. Front Immunol (2017) 8:550. doi: 10.3389/fimmu.2017.00550 28553293 PMC5427094

[11] Hoepel W Chen HJ Geyer CE Allahverdiyeva S Manz XD de Taeye SW. High titers and low fucosylation of early human anti-Sars-Cov-2 igg promote inflammation by alveolar macrophages. Sci Transl Med (2021) 13(596):eabf8654. doi: 10.1126/scitranslmed.abf8654 33979301 PMC8158960

[12] Marklund E Leach S Axelsson H Nyström K Norder H Bemark M. Serum-igg responses to sars-Cov-2 after mild and severe covid-19 infection and analysis of igg non-responders. PloS One (2020) 15(10):e0241104. doi: 10.1371/journal.pone.0241104 33085715 PMC7577439

[13] Kurashima K Kagiyama N Ishiguro T Takaku Y Nakajima H Shibata S. Igg antibody seroconversion and the clinical progression of covid-19 pneumonia: A retrospective, cohort study. medRxiv (2020). doi: 10.1101/2020.07.16.20154088

[14] Vidarsson G Dekkers G Rispens T. Igg subclasses and allotypes: From structure to effector functions. Front Immunol (2014) 5. doi: 10.3389/fimmu.2014.00520 PMC4202688 25368619

[15] Cobb BA. The history of igg glycosylation and where we are now. Glycobiology (2020) 30(4):202–13. doi: 10.1093/glycob/cwz065 PMC7109348 31504525

[16] Shields RL Lai J Keck R O'Connell LY Hong K Meng YG. Lack of fucose on human Igg1 n-linked oligosaccharide improves binding to human fcγriii and antibody-dependent cellular toxicity. J Biol Chem (2002) 277(30):26733–40 doi: 10.1074/jbc.M202069200. 11986321

[17] Pongracz T Vidarsson G Wuhrer M. Antibody glycosylation in covid-19. Glycoconjugate J (2022) 39:335–44. doi: 10.1007/s10719-022-10044-0. PMC8799414 35091890

[18] Larsen MD de Graaf EL Sonneveld ME Plomp HR Nouta J Hoepel W. Afucosylated igg characterizes enveloped viral responses and correlates with covid-19 severity. Science (2021) 371(6532):eabc8378. doi: 10.1126/science.abc8378 33361116 PMC7919849

[19] Chakraborty S Gonzalez J Edwards K Mallajosyula V Buzzanco AS Sherwood R. Proinflammatory igg fc structures in patients with severe covid-19. Nat Immunol (2021) 22(1):67–73. doi: 10.1038/s41590-020-00828-7 33169014 PMC8130642

[20] Chakraborty S Gonzalez JC Sievers BL Mallajosyula V Chakraborty S Dubey M. Early non-neutralizing, afucosylated antibody responses are associated with covid-19 severity. Sci Transl Med (2022) 14(635):eabm7853. doi: 10.1126/scitranslmed.abm7853 35040666 PMC8939764

[21] Wang Taia T Sewatanon J Memoli Matthew J Wrammert J Bournazos S Bhaumik Siddhartha K. Igg antibodies to dengue enhanced for fcγriiia binding determine disease severity. Science (2017) 355(6323):395–8. doi: 10.1126/science.aai8128 PMC5557095 28126818

[22] Larsen MD Lopez-Perez M Dickson EK Ampomah P Tuikue Ndam N Nouta J. Afucosylated plasmodium falciparum-specific igg is induced by infection but not by subunit vaccination. Nat Commun (2021) 12(1):5838. doi: 10.1038/s41467-021-26118-w 34611164 PMC8492741

[23] Ackerman ME Crispin M Yu X Baruah K Boesch AW Harvey DJ. Natural variation in fc glycosylation of hiv-specific antibodies impacts antiviral activity. J Clin Invest (2013) 123(5):2183–92. doi: 10.1172/JCI65708 PMC3637034 23563315

[24] Kapur R Kustiawan I Vestrheim A Koeleman CAM Visser R Einarsdottir HK. A prominent lack of Igg1-fc fucosylation of platelet alloantibodies in pregnancy. Blood (2014) 123(4):471–80. doi: 10.1182/blood-2013-09-527978 PMC3901064 24243971

[25] Van Coillie J Pongracz T Rahmöller J Chen H-J Geyer C van Vlught LA. The Bnt162b2 mrna sars-Cov-2 vaccine induces transient afucosylated Igg1 in naive but not antigen-experienced vaccinees. bioRxiv (2022). doi: 10.1101/2022.02.14.480353 PMC9756879 36529104

[26] Petrović T Alves I Bugada D Pascual J Vučković F Skelin A. Composition of the immunoglobulin G glycome associates with the severity of covid-19. Glycobiology (2021) 31(4):372–7. doi: 10.1093/glycob/cwaa102 PMC7717252 33174592

[27] Hou H Yang H Liu P Huang C Wang M Li Y. Profile of immunoglobulin G n-glycome in covid-19 patients: A case-control study. Front Immunol (2021) 12:748566. doi: 10.3389/fimmu.2021.748566 34630427 PMC8495247

[28] Vicente MM Alves I Gaifem J Rodrigues CS Fernandes Â Dias AM. Altered igg glycosylation at covid-19 diagnosis predicts disease severity. Eur J Immunol (2022) 52(6):946–57. doi: 10.1002/eji.202149491 PMC9087392 35307819

[29] Pongracz T Nouta J Wang W van Meijgaarden KE Linty F Vidarsson G. Immunoglobulin G1 fc glycosylation as an early hallmark of severe covid-19. EBioMedicine (2022) 78:103957. doi: 10.1016/j.ebiom.2022.103957 35334306 PMC8938159

[30] Trapé ÁA Camacho-Cardenosa M Camacho-Cardenosa A Merellano-Navarro E Rodrigues JAL da Silva Lizzi EA. Effects of moderate-intensity intermittent hypoxic training on health outcomes of patients recovered from covid-19: The aerobicovid study protocol for a randomized controlled trial. Trials (2021) 22(1):534. doi: 10.1186/s13063-021-05414-2 34384461 PMC8358903

[31] Brouwer PJ Caniels TG van der Straten K Snitselaar JL Aldon Y Bangaru S. Potent neutralizing antibodies from covid-19 patients define multiple targets of vulnerability. Science (2020) 369(6504):643–50. doi: 10.1126/science.abc5902 PMC7299281 32540902

[32] Falck D Jansen BC Haan Nd Wuhrer M. High-throughput analysis of igg fc glycopeptides by lc-Ms. High-Throughput Glycomics Glycoproteomics Springer (2017) 31–47. doi: 10.1007/978-1-4939-6493-2_4 27743357

[33] Jansen BC Falck D de Haan N Hipgrave Ederveen AL Razdorov G Lauc G. Lacytools: A targeted liquid chromatography–mass spectrometry data processing package for relative quantitation of glycopeptides. J Proteome Res (2016) 15(7):2198–210. doi: 10.1021/acs.jproteome.6b00171 27267458

[34] Perkovic MN Bakovic MP Kristic J Novokmet M Huffman JE Vitart V. The association between galactosylation of immunoglobulin G and body mass index. Prog Neuropsychopharmacol Biol Psychiatry (2014) 48:20–5. doi: 10.1016/j.pnpbp.2013.08.014 24012618

[35] Petrović T Vijay A Vučković F Trbojević-Akmačić I Ollivere BJ Marjanović D. Igg n-glycome changes during the course of severe covid-19: An observational study. eBioMedicine (2022) 81:104101. doi: 10.1016/j.ebiom.2022.104101 35773089 PMC9234382

[36] Lippold S Nicolardi S Domínguez-Vega E Heidenreich A-K Vidarsson G Reusch D. Glycoform-resolved fcɣriiia affinity chromatography–mass spectrometry. mAbs (2019) 11(7):1191–6. doi: 10.1080/19420862.2019.1636602 PMC6748599 31276431

[37] Gudelj I Lauc G Pezer M. Immunoglobulin G glycosylation in aging and diseases. Cell Immunol (2018) 333:65–79. doi: 10.1016/j.cellimm.2018.07.009 30107893

[38] Karsten CM Pandey MK Figge J Kilchenstein R Taylor PR Rosas M. Anti-inflammatory activity of Igg1 mediated by fc galactosylation and association of fcγriib and dectin-1. Nat Med (2012) 18(9):1401–6. doi: 10.1038/nm.2862 PMC3492054 22922409

[39] van Osch TLJ Nouta J Derksen NIL van Mierlo G van der Schoot CE Wuhrer M. Fc galactosylation promotes hexamerization of human Igg1, leading to enhanced classical complement activation. J Immunol (2021) 207(6):1545–54. doi: 10.4049/jimmunol.2100399 PMC8428746 34408013

[40] Potere N Batticciotto A Vecchié A Porreca E Cappelli A Abbate A. The role of il-6 and il-6 blockade in covid-19. Expert Rev Clin Immunol (2021) 17(6):601–18. doi: 10.1080/1744666X.2021.1919086 33874829

[41] Darif D Hammi I Kihel A El Idrissi Saik I Guessous F Akarid K. The pro-inflammatory cytokines in covid-19 pathogenesis: What goes wrong? Microbial Pathogenesis (2021) 153:104799. doi: 10.1016/j.micpath.2021.104799 33609650 PMC7889464

[42] Štambuk J Nakić N Vučković F Pučić-Baković M Razdorov G Trbojević-Akmačić I. Global variability of the human igg glycome. Aging (Albany NY) (2020) 12(15):15222–59. doi: 10.18632/aging.103884 PMC7467356 32788422

[43] de Jong SE Selman MHJ Adegnika AA Amoah AS van Riet E Kruize YCM. Igg1 fc n-glycan galactosylation as a biomarker for immune activation. Sci Rep (2016) 6(1):28207. doi: 10.1038/srep28207 27306703 PMC4910062

