# Supplementary material for: The IgG glycome of SARS-CoV-2 infected individuals reflects disease course and severity
Source: Front Immunol. 2022 Oct 18;13:993354. doi: 10.3389/fimmu.2022.993354 (PMC9641981; doi:10.3389/fimmu.2022.993354)
Supplement: Supplementary file 1 [file DataSheet_1.pdf]

Supplementary Figures

Table of contents

Supplementary Figure 1: Comparison of IgG1 glycosylation between the Brazilian and German cohorts.

Supplementary Figure 2: IgG1 glycosylation in patients over time with individual datapoints shown.

Supplementary Figure 3: Confounding effects of BMI on IgG1 glycosylation.

Supplementary Figure 4: Total IgG1 glycosylation compared between inpatients and outpatients, adjusted for the days since onset of symptoms.

Supplementary Figure 5: Comparison of IgG1 glycosylation between ICU and non-ICU patients.

Supplementary figure 6: Correlation graphs showing the significant Spearman's correlations between IgG1 glycosylation and inflammatory markers.

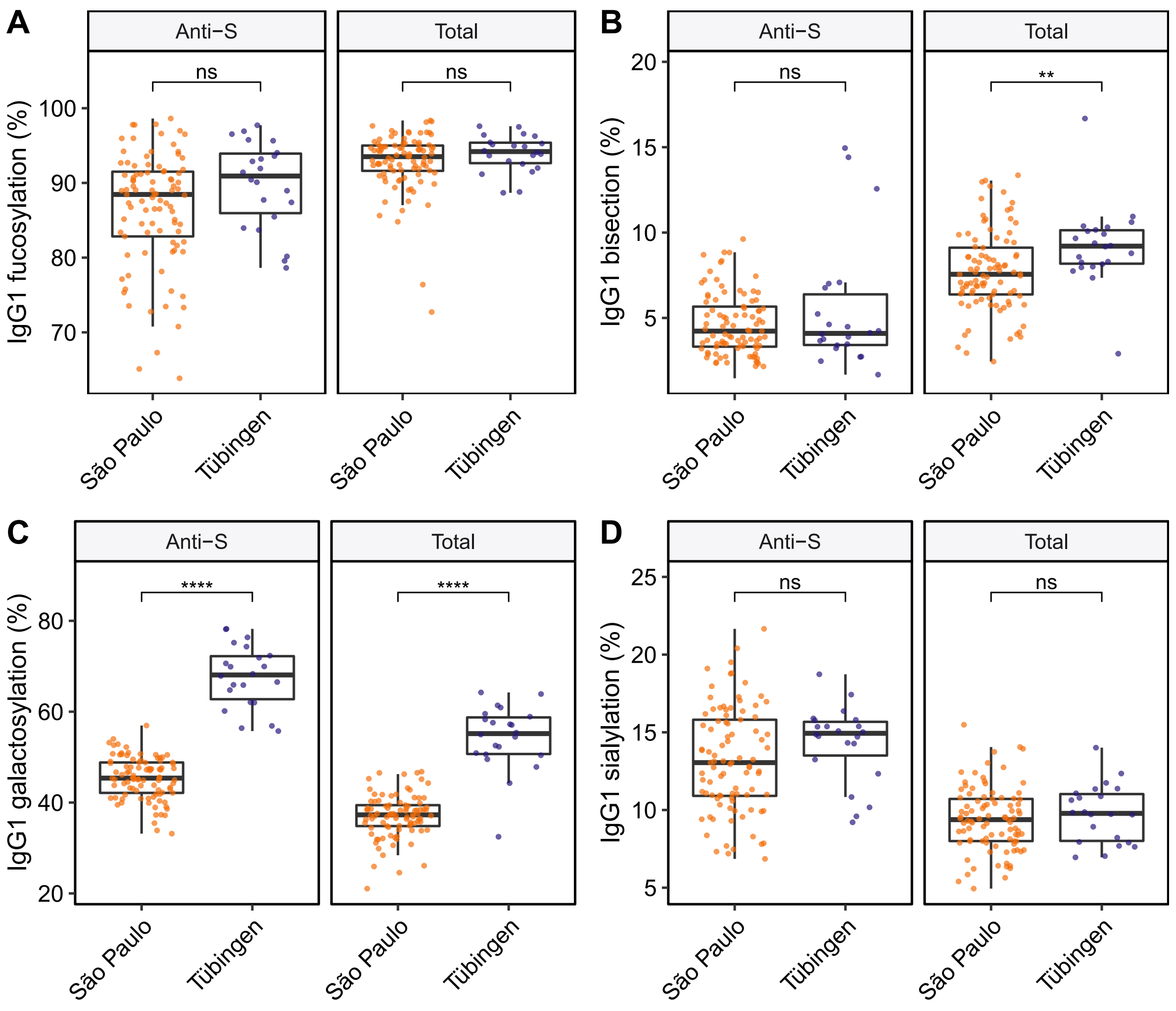

Figure S1: **IgG1 glycosylation differs between Brazilian (São Paulo) and German (Tübingen) COVID-19 patients.** For the glycosylation traits fucosylation (**A**), bisection (**B**), galactosylation (**C**) and sialylation (**D**) anti-S (left) and total (right) IgG1 glycosylation was compared between COVID-19 patients from the São Paulo (n = 93) and Tübingen (n = 22) cohorts. Only baseline samples from inpatients and outpatients are included. Significance levels shown are based on the *p*-values from Wilcoxon rank sum tests. *, **, ****: *p*-value < 0.05, 0.01, 0.0001, respectively. ns: not significant (*p*-value ≥ 0.05).

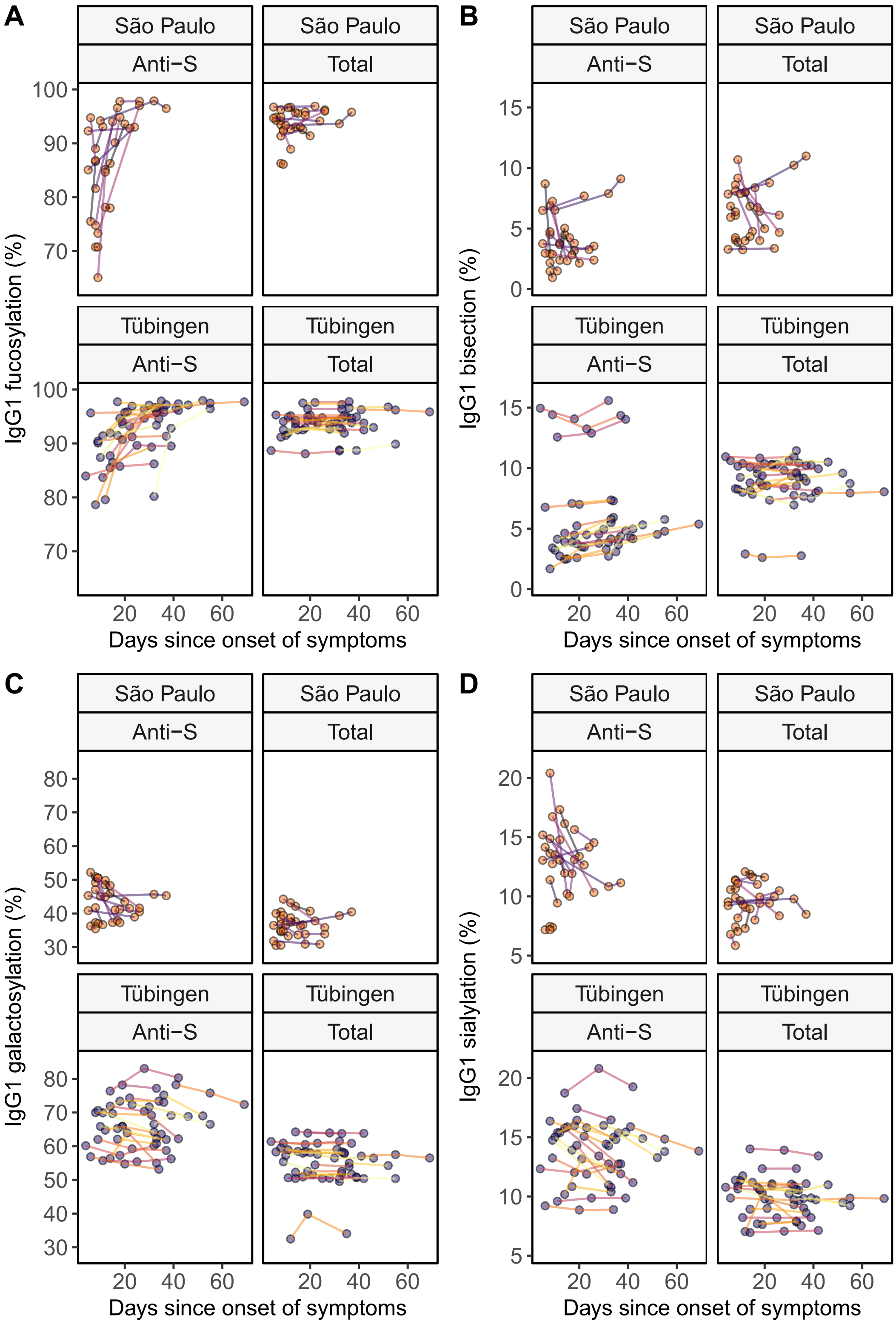

Figure S2: Total and anti-S IgG1 glycosylation in patients over time with individual datapoints shown. For the glycosylation traits fucosylation (**A**), bisection (**B**), galactosylation (**C**) and sialylation (**D**) both anti-S (left) and total (right) IgG1 glycosylation in in- and outpatients from the São Paulo (top, n = 15) and Tübingen (bottom, n = 20) cohorts are plotted against the days since onset of symptoms. Samples that originate from the same patient are connected with lines.

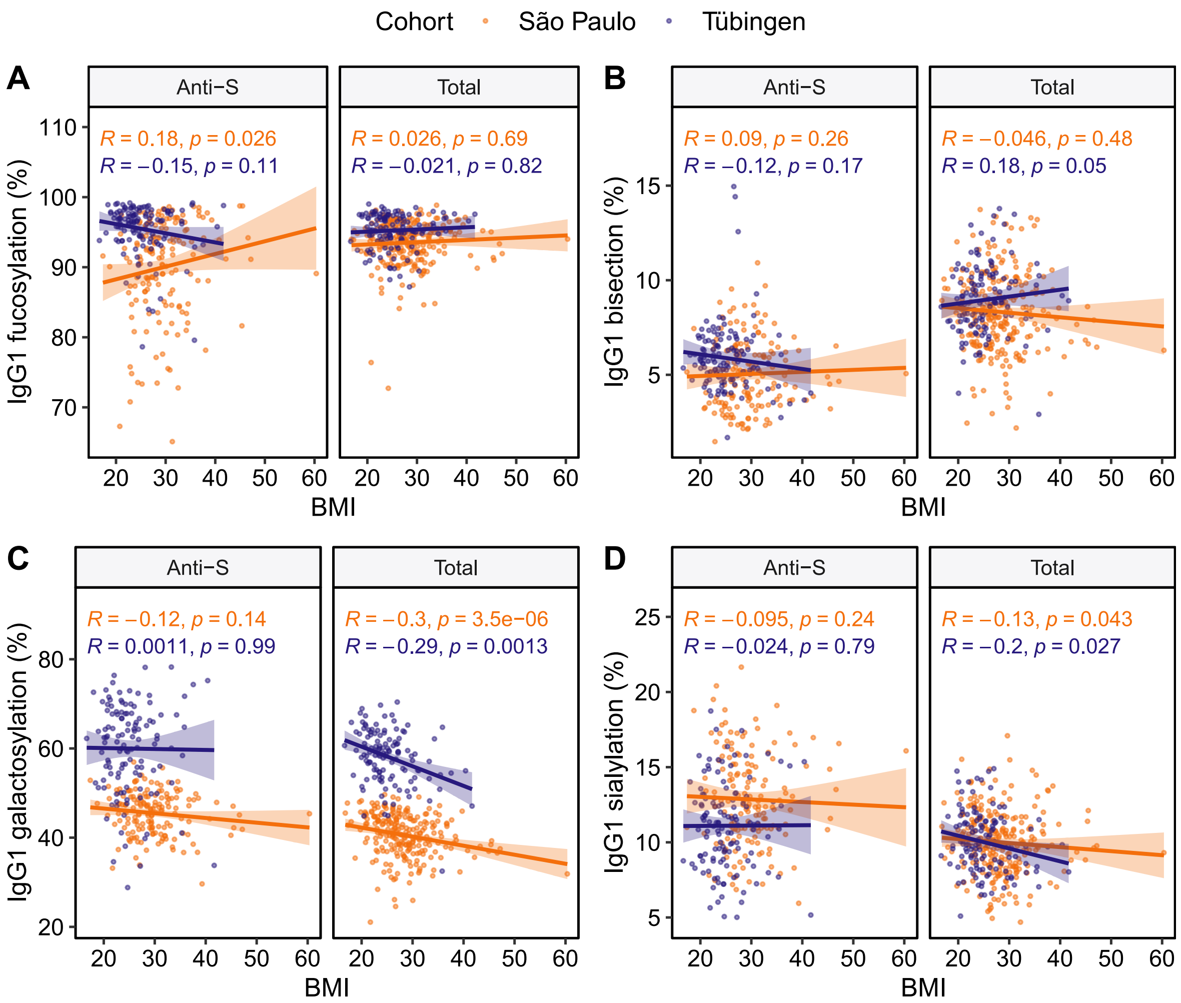

Figure S3: Correlations between total and anti-S IgG1 Fc glycosylation and BMI. For both anti-S (left) and total (right) IgG1 the derived glycosylation traits fucosylation (**A**), bisection (**B**), galactosylation (**C**) and sialylation (**D**) were plotted against BMI. Spearman's correlation coefficients (*R*) with corresponding *p*-values are shown separately for the São Paulo cohort (orange) and the Tübingen cohort (purple). To visualize the trends in each cohort, linear regression lines with 95% confidence intervals were added. Total IgG1 galactosylation showed significant negative correlations with BMI in both cohorts.

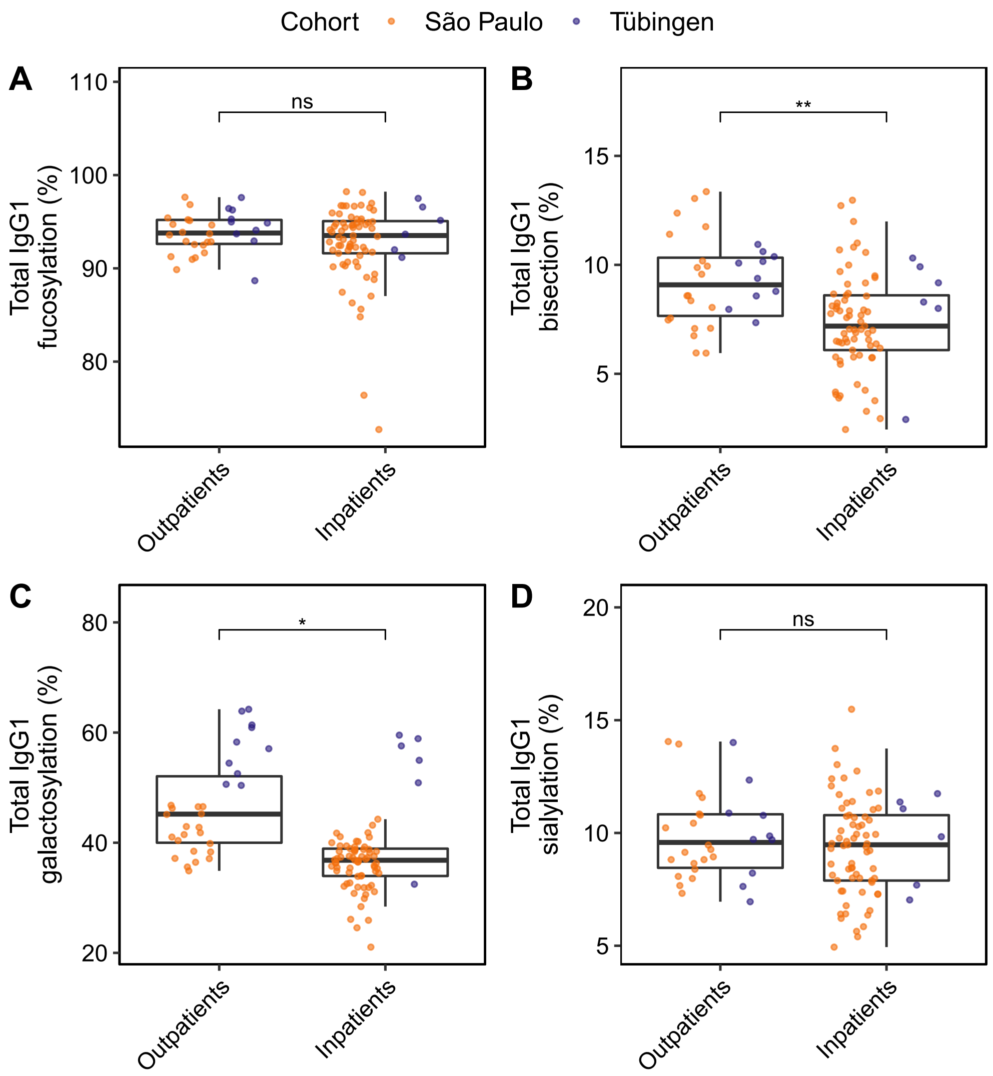

Figure S4: **Total IgG1 glycosylation in inpatients compared to outpatients, adjusted for days since onset of symptoms.** For the derived glycosylation traits fucosylation (**A**), bisection (**B**), galactosylation (**C**) and sialylation (**D**) total IgG1 glycosylation was compared between outpatients (n = 30) and inpatients (n = 73). The significance levels shown are based on the *p*-values of the coefficients for the glycosylation traits in the logistic regression models with adjustment for the effects of age, sex, BMI, the cohort and the interaction between age and sex. ***, ****: *p*-value < 0.001, 0.0001 and ns: not significant (*p*-value ≥ 0.05)

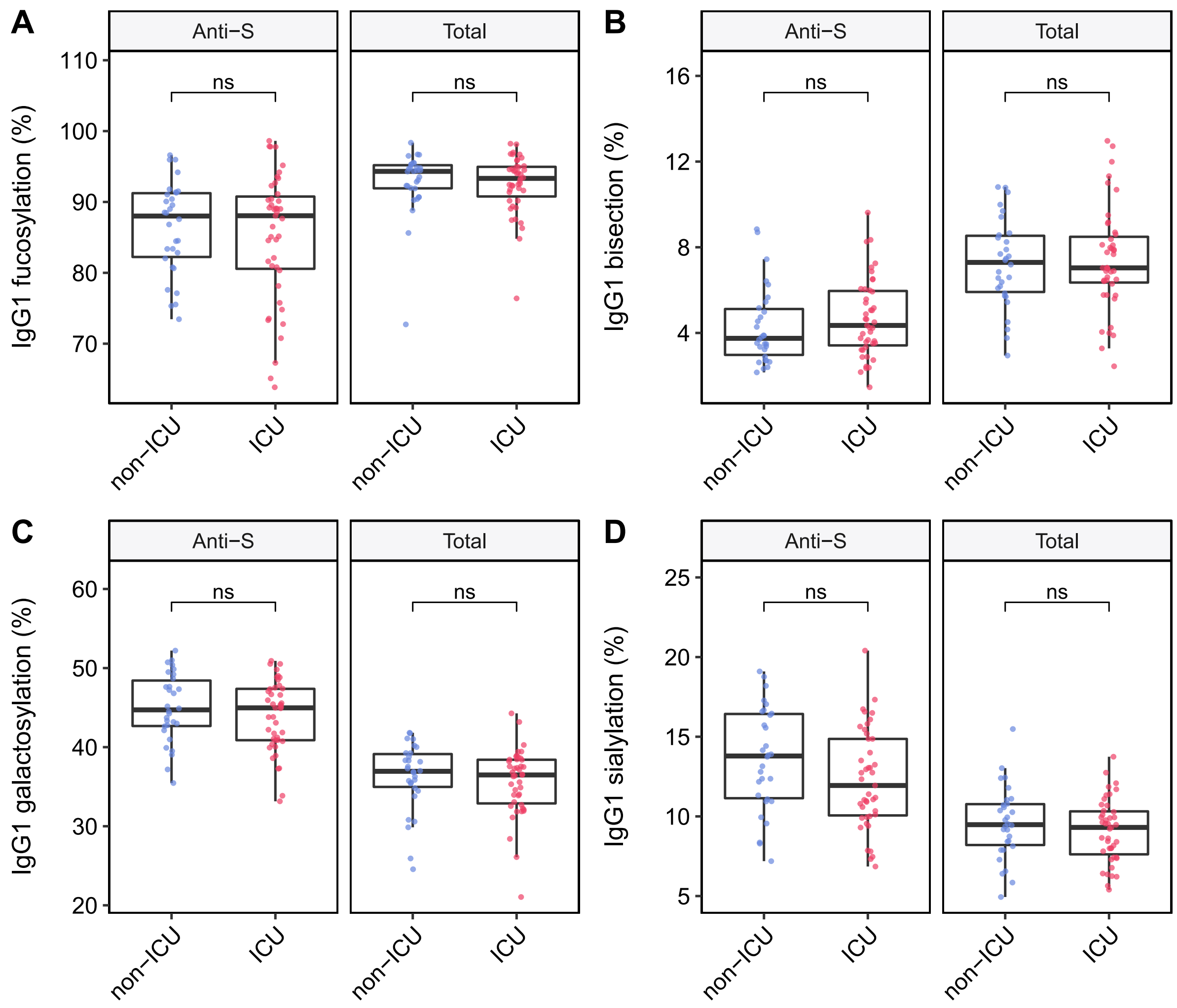

Figure S5: Total and anti-S IgG1 Fc glycosylation are not significantly different in patients that have been admitted to an intensive care unit (ICU) compared to patients that have not been admitted to an ICU (non-ICU). For the derived glycosylation traits fucosylation (**A**), bisection (**B**), galactosylation (**C**) and sialylation (**D**) both total and anti-S IgG1 glycosylation was compared between ICU (n = 43) and non-ICU (n = 30) patients from the São Paulo cohort. The significance levels shown are based on the *p*-values of the coefficients for the glycosylation traits in the logistic regression models with adjustment for the effects of age, sex, BMI, the days since onset of symptoms and the interaction between age and sex. Ns: not significant (*p*-value ≥ 0.05).

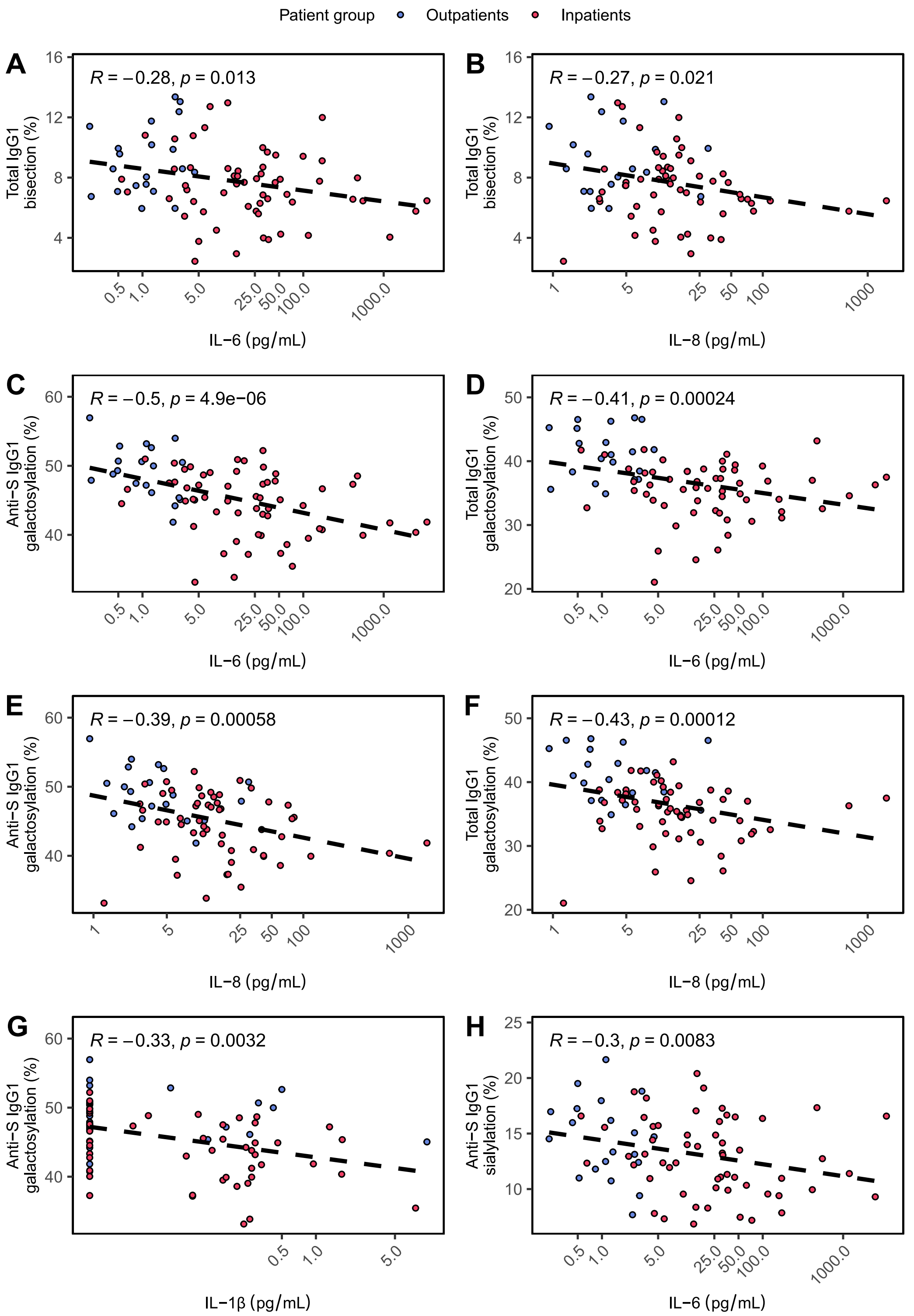

Figure S6: Correlation graphs showing significant Spearman's correlations between glycosylation traits and cytokine concentrations at baseline. Glycosylation traits were plotted against the cytokine concentrations in 57 in- and 20 outpatients. Cytokines were measured in samples collected at the same timepoint as the samples used for determining IgG1 Fc *N*-glycosylation profiles and infection status for SARS-CoV-2. Spearman's correlation coefficients (*R*) are shown with corresponding *p*-values. Only the combinations of glycosylation traits and cytokines are shown for which the Spearman's correlation was significant (*p*-value ≤ 0.05).
